# Robotic arm-assisted total hip arthroplasty for preoperative planning and intraoperative decision-making

**DOI:** 10.1186/s13018-023-04095-8

**Published:** 2023-08-21

**Authors:** Hanpeng Lu, Qiang Xiao, Hong Xu, Tingfang Yan, Zongke Zhou

**Affiliations:** 1 Department of Orthopaedic Surgery, West China Hospital, Sichuan University, No. 37, Guoxue Road, Wuhou District, Chengdu, 610041 Si Chuan People’s Republic of China; 2 Yuanhua Intelligent Technology Co., Ltd, Shenzhen, People’s Republic of China

**Keywords:** Total hip arthroplasty, Robot-assisted surgery, Preoperative planning, Intraoperative decision-making

## Abstract

**Aims:**

This article aimed to explore the efficacy of robotic arm-assisted total hip arthroplasty (THA) in improving preoperative planning and intraoperative decision-making.

**Methods:**

In this single-center, prospective, randomized clinical controlled trial, 60 patients were randomly divided into two groups: conventional THA (cTHA) and robotic arm-assisted THA (rTHA). The rTHA underwent procedures using a robot-assisted surgical system, which generated three-dimensional models to determine the most appropriate prosthesis size and position. The standard process of replacement was executed in cTHA planned preoperatively via X-ray by experienced surgeons. Differences between predicted and actual prosthetic size, prosthetic position, and leg length were evaluated.

**Results:**

Sixty patients were included in the study, but one patient was not allocated due to anemia. No significant preoperative baseline data difference was found between the two groups. The actual versus predicted implantation size of both groups revealed that 27/30 (90.0%) in the rTHA group and 25/29 (86.2%) in the cTHA group experienced complete coincidence. The coincidence rate for the femoral stem was higher in the rTHA group (83.3%) than that in the cTHA group (62.7%). Between the actual and predicted rTHA, the difference in anteversion/inclination degree (< 6°) was largely dispersed, while cTHA was more evenly distributed in degree (< 9°). The differences in leg length between the surgical side and contralateral side showed a significant deviation when comparing the two groups (*P* = 0.003), with 0.281 (− 4.17 to 3.32) mm in rTHA and 3.79 (1.45–6.42) mm in cTHA.

**Conclusion:**

Robotic arm-assisted total hip arthroplasty can be valuable for preoperative planning and intraoperative decision-making.

## Introduction

Total hip arthroplasty (THA) is an effective method for advanced hip joint diseases. At present, the main purpose of THA is to reduce pain and improve the function of the hip joint. Globally, there are more than 1 million THAs every year, and the number is increasing yearly [1]. Despite the improvement of prosthesis design, friction interface and coating materials, the demand for THA revision has increased by more than 20% in the past 15 years and is expected to double in the next 10 years [2]. Aseptic loosening, periprosthetic fracture, postoperative dislocation, limb length discrepancy (LLD), and infection constitute the main causes of revision after THA [3].

With the increasing types of prostheses, THA has significantly grown in complexity over time. Inappropriate placement will not only affect the stability and bone integration and increase the probability of complications after THA [4] but also lead to biomechanical changes and affect the survival time of the prosthesis [5]. Furthermore, inappropriate placement will lead to LLD after THA, affecting the postoperative function of the patients [6]. Accurate preoperative planning is an important step to obtain a satisfactory prognosis and reduce complications, including the right size of the prosthesis, appropriate height of the neck osteotomy and suitable depth of acetabular reaming before THA [7–9]. Therefore, as a part of the overall evaluation, preoperative planning is an indispensable part of THA.

The X-ray template or digital two-dimensional template is a commonly used method for preoperative planning. It predicts the size and position of the prosthesis with an accuracy of 78–95% [10–15] and the appropriate length of the lower extremities. However, the use of a magnification marker with digital radiographs for preoperative templating is generally deemed as inaccurate [16], and the projection angle of the pelvis and femur is not standard [17, 18], resulting in a high incidence of complications after THA [19]. Preoperative planning based on 3D reconstruction computed tomography (CT) is more accurate and can effectively reduce the learning curve [20], but its own planning process is challenging for doctors. In addition, successful surgery needs to reproduce planning results perfectly, and preoperative planning is difficult to strictly carry out due to the lack of real-time guidance during the operation [21]. The robot-assisted system for THA, used in this study, is composed of a planning navigation system and manipulator control system. It can not only carry out accurate preoperative planning but also realize intraoperative information interaction between the operator and the computer, which shows substantial practical value and application prospects [22]. However, the effect of preoperative planning and intraoperative decision-making of the robot-assisted system for THA still needs to be further verified.

In this study, an independently developed manipulator-assisted robot was used for preoperative planning and compared with conventional two-dimensional digital X-ray planning. The purpose of this study was to focus on whether robotic arm-assisted THA (rTHA) can improve preoperative planning compared to conventional THA (cTHA). Besides, the improvement of intraoperative decision-making between the two groups was also explored in this study.

## Materials and methods

**Fig. 1:**
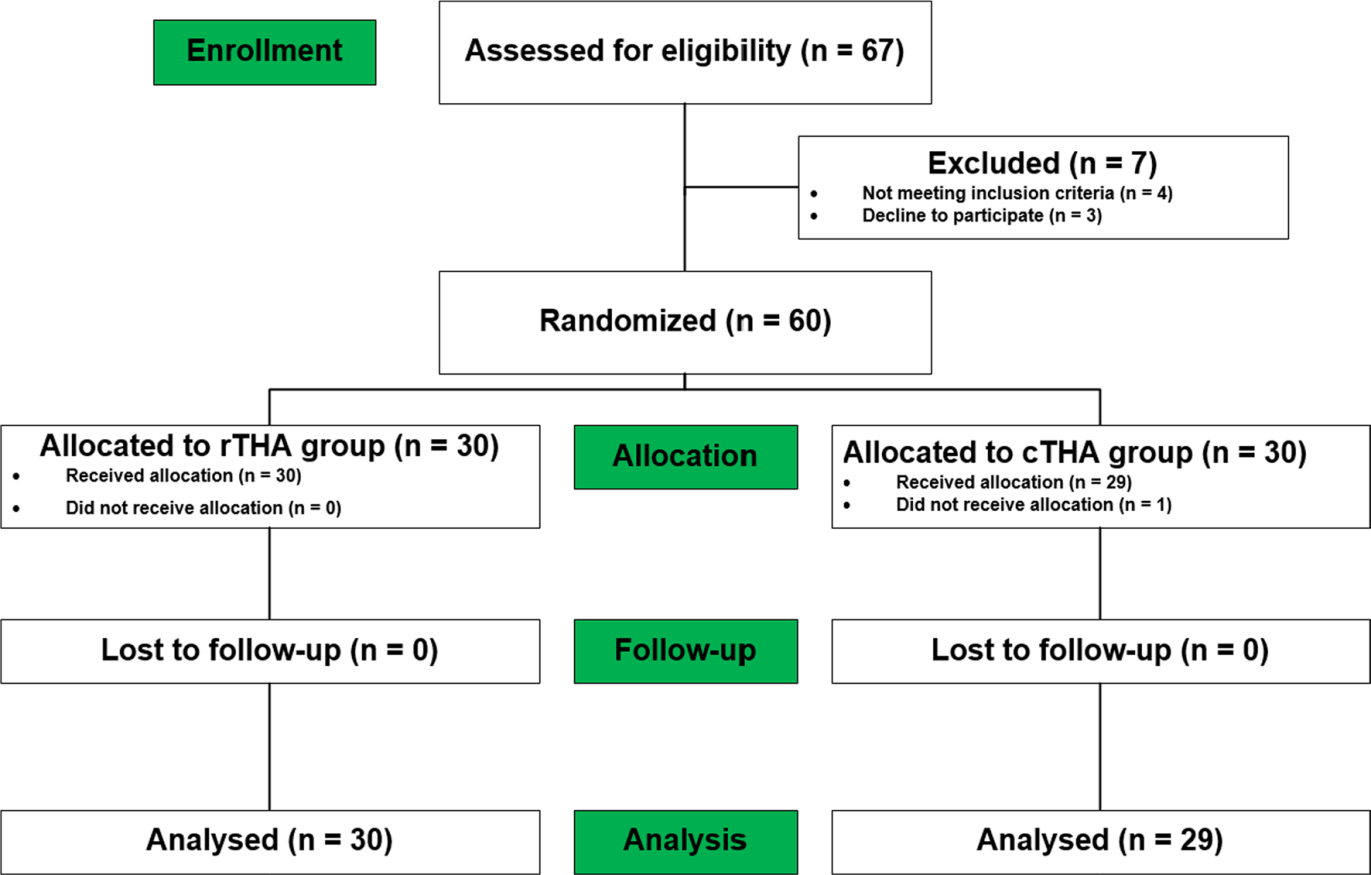
Consolidated standards of reporting trial (consort) diagram showing the flow of patients through robotic arm-assisted total hip arthroplasty (rTHA) versus conventional total hip arthroplasty (cTHA)

This study was a single-center (Orthopedics, West China Hospital, Chengdu, China), prospective, randomized clinical controlled trial including participants aged 18–80 years old. This trial was approved by the Ethics Committee on Clinical Trials, West China Hospital of Sichuan University (HX-IRB-AF-12-V4.0). All patients provided written informed consent before participation. The study was registered in the Clinical Trial Registry (ChiCTR2200059968). The exclusion criteria were as follows: neuromuscular dysfunction, including paralysis, myolysis or abductor weaknes; active infection lesions; severe hip deformity and hip dysplasia with Cone grade 3 or 4; ankylosing spondylitis patients with bony ankylosis or severe stiffness; bilateral hip arthroplasty at the same time; severe internal and surgical diseases or weak physique; and poor expected compliance. In total, 67 potential test persons were scheduled to undergo primary THA, where 4 patients were ineligible and 3 patients declined participation. The remaining 60 patients were randomized into two groups, while 1 patient in the cTHA group was finally not allocated due to anemia (Fig. 1).

### Randomization

The interactive network response system Clinflash IRT v2.2.10 (Clinflash Healthcare Technology, Jiaxing Clinflash Computer Technology Co. Ltd.) automatically distributed groups randomly according to the order of entry to reduce the test bias caused by sampling error. The random table was generated by the specified person, and the random table was generated by SAS version 9.4 (SAS Institute, Cary, NC) according to the preset number of seeds and the number of blocks. The ratio of the trial group to the control group was 1:1 by using the block randomization design according to central stratification. Any subjects who had completed randomization but withdrew from this clinical trial before the commencement of treatment were retained.

### Intervention

**Fig. 2:**
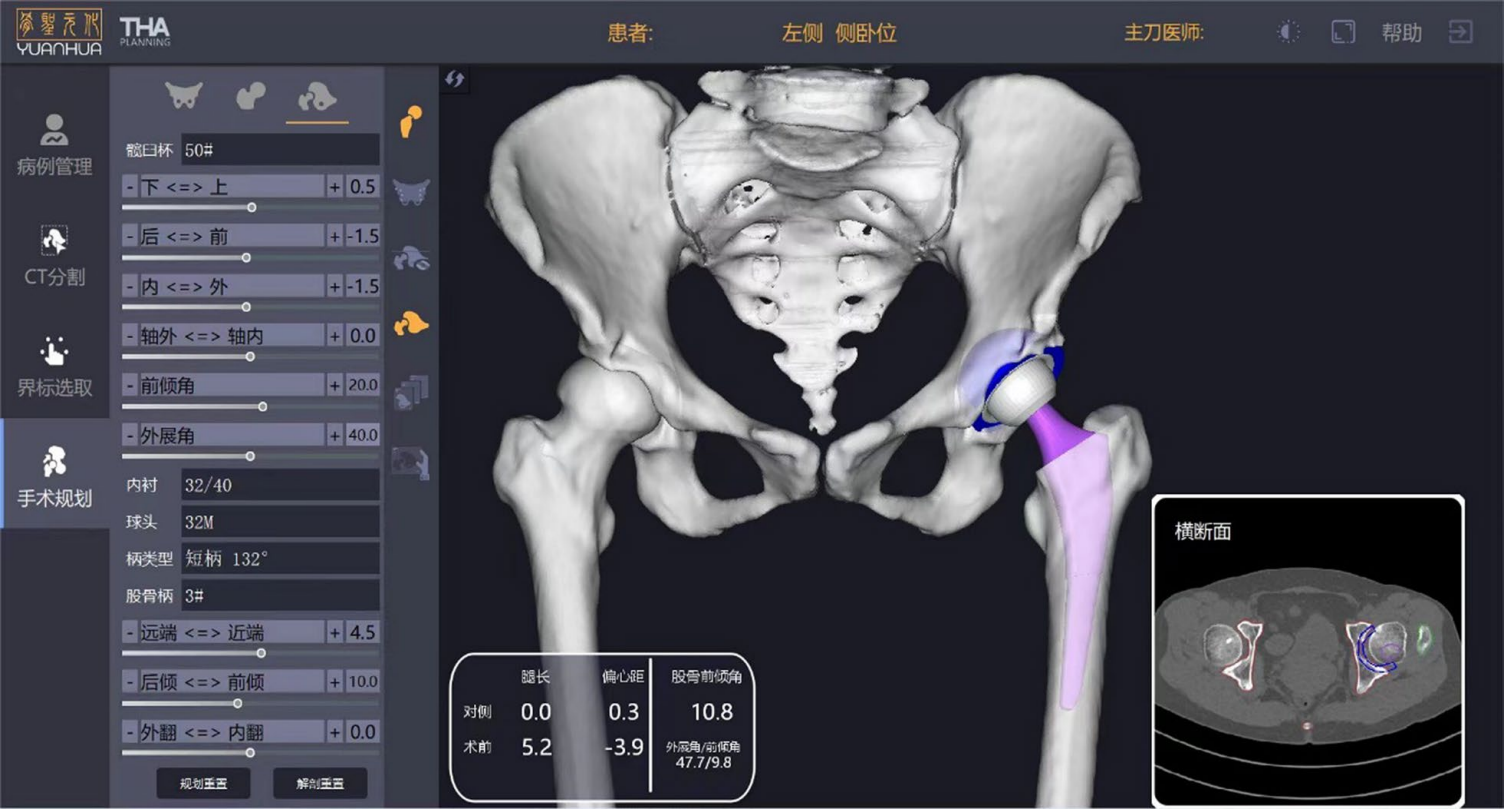
Preoperative planning with computer for robotic arm-assisted total hip arthroplasty. 手术规划, surgical planning; 髋臼杯, acetabular cup; 内衬, acetabular liner; 球头, femoral head; 柄类型, stem type; 股骨柄, femoral stem

A total of 60 patients were randomly divided into two groups: patients who underwent conventional THA (cTHA group) and patients who underwent robotic arm-assisted THA (rTHA group). The patients in the cTHA group underwent preoperative planning, during which the pelvic digital X-ray (enlarged by 100%) was compared to a two-dimensional digital model of the prosthesis. Conversely, patients undergoing rTHA had preoperative CT scans of the pelvis and both knees, in which the pelvic CT was compared to a three-dimensional digital model of the prosthesis using a robotic navigation system for precise implant positioning (Fig. 2). Template assistance was utilized to restore the natural center of rotation and offset, with the opposite side serving as a reference, if normal, and to correct any discrepancies in leg length as much as possible. The planned positions of the acetabular components for both groups were 40° inclination and 20°–25° anteversion (anatomic angles for both rTHA group and cTHA group). The corrections were made to align the anterior superior iliac spines on the horizontal plane in order to correct for pelvic tilt. These adjustments were necessary due to pelvic deformity or improper positioning during the imaging process. All the X-rays and CT were acquired in a supine position. All rTHA procedures were performed using a single robot-assisted surgical system (YUANHUA-THA; Yuanhua Orthopaedic Robotics Limited, Shenzhen, China), which was a semiactive surgical robotic designed to assist for patients undergoing THA. During the surgery, the system can provide real-time feedback on the position of the acetabular cup (anatomic angles), and was used for surgical planning, navigation, precise bone resection, and implant placement. The surgical procedures were described in the technical manuals provided by the manufacturer. In the cTHA group, the standard process of replacement was executed and the surgeon positions the acetabular cup based on anatomical landmarks during the procedure. All patients underwent the posterolateral approach, and the cementless prosthesis (ceramics on ceramic interface) came from Zhengtian Medical Instrument (Tianjin, China).

### Outcome measures

**Fig. 3:**
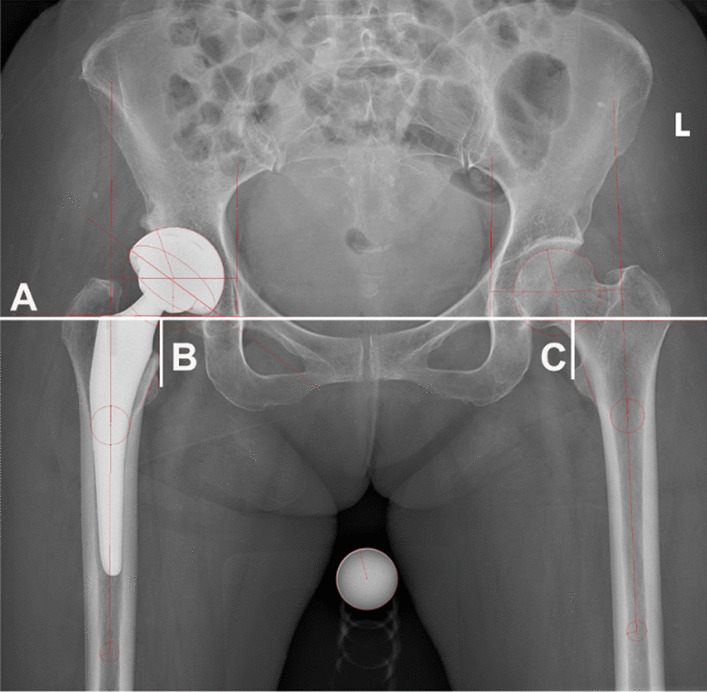
Presenting the process of calculating the length of both lower limbs. A. Bilateral tear drop line; B. Distance from the left lesser trochanter to the tear drop line; C. Distance from the right lesser trochanter to the tear drop line

All patients were examined by X-ray and CT within 14 days before surgery. One day before the operation, the planning for the cTHA group was based on X-ray measurements of the size and position of the prosthesis and the length of the lower extremities. The length was determined by drawing a horizontal reference line through bilateral tears and measuring the distance from the vertex of the surgical and contralateral lesser trochanter to the horizontal reference line (Fig. 3). On the first day after the operation, a CT was performed and the anteversion/inclination angles of the acetabular cup were measured with Mimics 24.0 software (Materialise, Leuven, Belgium) according to Murray's definition of the anatomical acetabular angle referenced to the anterior pelvic plane [23, 24]. The acceptable range was the planned angle ± 6°. After the surgical procedure, the leg length discrepancy (LLD) was assessed by measuring the vertical distance between the bilateral lesser trochanter and bilateral teardrop marks on the pelvis using anterior–posterior pelvic X-ray images. An LLD exceeding 10 mm is considered to be beyond the acceptable range [25]. The length of the lower extremities was measured by two independent researchers. When the difference between the two researchers was small (≤ 2 mm), the final results were averaged. When the difference between the two researchers was large (> 2 mm), a third independent evaluation researcher was introduced. The average values of the two most similar results were taken as the final results. The robotic intraoperative guidance capability was assessed by the actual versus predicted anteversion/inclination of the acetabular cup and the postoperative difference in bilateral leg lengths.

### Statistical analysis

SPSS 27.0 (IBM, Chicago, IL, USA) was used for data analysis. Data were reported as the means and standard deviations or as proportions. The independent-samples t-test was used for continuous data showing a normal distribution, the Mann‒Whitney U test was used for continuous data showing skew, and the chi-squared test or Fisher precision test was used for categorical data. A two-sided *P* value < 0.05 was considered significant. In this study, we utilized the independent-samples t-test to compare age and BMI. The Mann–Whitney U test was employed to evaluate limb-length inequality. The chi-squared test was applied to compare surgical side, gender, and the actual versus predicted size/degree of implant.

## Results

**Fig. 4:**
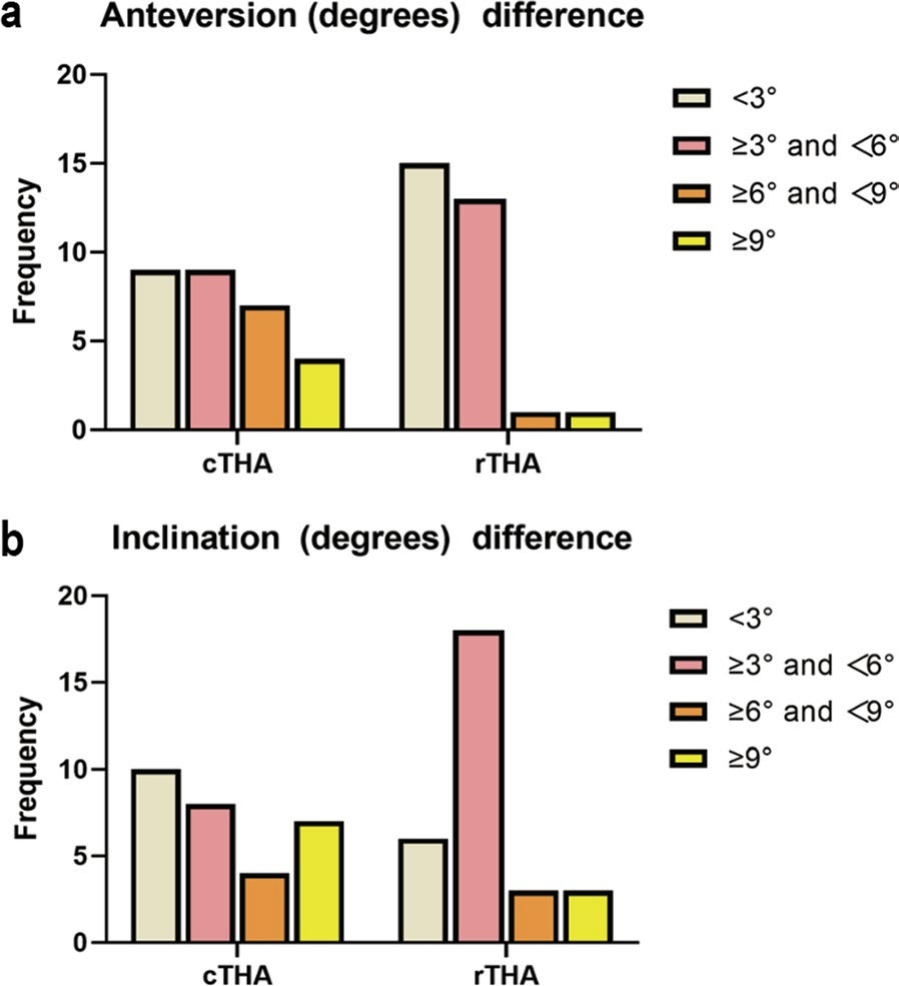
Actual versus predicted anteversion or inclination (degrees) of the acetabular cup. **a** Anteversion degree difference of acetabular cup; **b** inclination degree difference of acetabular cup

A total of 60 patients were included in our study. Fifty-nine patients were analyzed, while 1 patient in the cTHA group did not receive allocation due to anemia. There was no significant difference in all preoperative baseline data in Table 1 between the two groups although the difference in BMI was close to be significant (*P* = 0.058). The results of actual implantation size versus predicted value are shown in Table 2. The complete coincidence rate of prosthesis size between preoperative planning and the actual acetabular cup in the rTHA group was 27/30 (90.0%), and that in the cTHA group was 25/29 (86.2%). For the neck shaft angle, the complete coincidence rate in the rTHA group was 29/30 (96.7%), which was similar to that in the cTHA group (28/29, 96.6%). The complete coincidence rate between preoperative planning and the actual femoral stem in the rTHA group was 25/30 (83. 3%), which was higher than that in the cTHA group (18/29, 62.7%). Actual versus predicted anteversion/inclination (degrees) of the acetabular cup are shown in anteversion/inclination (degrees) differences in Table 3. The difference in cup anteversion (degree) for rTHA was mostly located in the group (< 3°), accounting for 15/30 (50%) of the cases. The cTHA group showed the largest number in the two groups (< 3°; ≥ 3° and < 6°), accounting for 9/29 (31.0%). In the group (> 9°), only 1/30 (3.3%) was in rTHA, while cTHA was higher, accounting for 4/29 (13.8%). The difference in cup inclination (degrees) showed a similar trend. The rTHA cases were mostly located in the ≥ 3° and < 6° group, accounting for 18/30 (60%), while the cTHA cases were more widely distributed across all four groups. The highest in group (< 3°) accounted for 10/29 (34.5%) and the group (> 9°) still accounted for 7/29 (24.1%) in cTHA. Next, we used 3 degrees as the distinguishing interval to intuitively describe these degree differences (Fig. 4). In brief, the difference in anteversion/inclination in rTHA was located more often in groups (< 3°, ≥ 3° and < 6°), while cTHA was more evenly distributed across group.

**Table 1:** Preoperative demographic data of analyzed patients

Variables	rTHA	cTHA	*P* value
Number	30	29	
Age	56.00 ± 12.33	56.52 ± 11.93	0.871
Sex, *n* (%)			0.329
Male	13 (43.3)	9 (31.0)	
Female	17 (56.7)	20 (69.0)	
Surgical side, *n* (%)			0.902
Left	16 (53.3)	15 (51.7)	
Right	14 (46.7)	14 (48.3)	
BMI (kg/m)^2^	24.26 ± 3.39	22.62 ± 3.12	0.058

*rTHA* robotic-assisted total hip arthroplasty, *cTHA* conventional total hip arthroplasty, *BMI* body mass index

**Table 2:** Actual implant size versus predicted implant size in this trial

Implant differential	rTHA	cTHA	*P* value
Size (acetabular cup)	27/30 (90.0%)	25/29 (86.2%)	0.962
Neck shaft angle	29/30 (96.7%)	28/29 (96.6%)	1.000
Size (Femoral stem)	25/30 (83. 3%)	18/29 (62.7%)	0.066

**Table 3:** Actual versus predicted anteversion or inclination (degrees) of the acetabular cup in this trial

	rTHA	cTHA	*P* value
*(a) Anteversion difference of acetabular cup between actual and predicted degrees*			
Anteversion (degrees) difference			
< 3°	15/30 (50.0%)	9/29 (31.0%)	0.138
≥ 3°and < 6°	13/30 (43.3%)	9/29 (31.0%)	0.329
≥ 6°and < 9°	1/30 (3.3%)	7/29 (24.1%)	0.051
≥ 9°	1/30 (3.3%)	4/29 (13.8%)	0.330
*(b) Inclination difference of acetabular cup between actual and predicted degrees*			
Inclination (degrees) difference			
< 3°	6/30 (20.0%)	10/29 (34.5%)	0.211
≥ 3°and < 6°	18/30 (60.0%)	8/29 (27.6%)	0.012
≥ 6°and < 9°	3/30 (10.0%)	4/29 (13.8%)	0.962
≥ 9°	3/30 (10.0%)	7/29 (24.1%)	0.271

In the process of surgical planning (Table 4), the difference between the planned length of surgical side and preoperative length of surgical side was 5.95 (3.48–8.65) mm for rTHA and was 2.90 (− 0.05 to 8.75) mm for cTHA (*P* = 0.275). Finally, the difference between postoperative length of surgical side and the length of opposite side showed a significant difference (*P* = 0.003), with 0.281 (− 4.17 to 3.32) mm in rTHA and 3.79 (1.45–6.42) mm in cTHA.

**Table 4:** Actual versus predicted leg length in this trial

Leg length difference	rTHA	cTHA	*P* value
Surgical side (planning vs. preoperative)	5.95 (3.48–8.65)	2.90 (− 0.05 to 8.75)	0.275
Surgical versus contralateral side (postoperative)	0.281 (− 4.17 to 3.32)	3.79 (1.45–6.42)	0.003

Surgical side (planning vs. preoperative): This refers to the discrepancy between the planned length of surgical side and preoperative length of surgical side

Surgical versus contralateral side (postoperative): This refers to the discrepancy between postoperative length of surgical side and the length of opposite side

## Discussion

In this prospective randomized clinical controlled trial, the purpose was to investigate the impact of preoperative planning and intraoperative decision-making caused by rTHA. Our results show that compared with cTHA, rTHA reduces leg length difference in lower limbs, and shows similar predictive effects of the size and position of the prosthesis as the experienced orthopedic surgeons, which reflects the relatively strong ability in preoperative planning and intraoperative decision-making.

An inaccurate size of the prosthesis can lead to serious intraoperative and postoperative complications and may even lead to the failure of THA [26]. For the cementless prosthesis, more than 2 mm of the interface between the prosthesis and bone will affect the stability [27]. If the size of the prosthesis is not appropriate, fretting will increase under the load of body weight prone to loosening, pain and other complications [28]. Therefore, it is necessary to select the appropriate prosthesis as much as possible to achieve matching between the prosthesis and the bone [29]. Detailed preoperative planning can accurately predict the size of the prosthesis and is also the premise of ideal mechanical conduction and long-term stability. In this study, the complete coincidence rate of the acetabular cup as preoperatively planned using the robotic system was 90.0%, which was slightly higher than that in cTHA (86.2%). At the same time, the complete coincidence rate of the femoral stem in rTHA was 83.3%, which was higher than that in the cTHA group (62.7%). On the one hand, it can be considered that the planning component of the robotic navigation system can have a stronger prediction ability than measurements on digital X-ray. Traditional X-ray only provides two-dimensional information, which cannot reflect information directly, such as the anteversion/inclination angle of the acetabular cup and bone mass of the acetabular wall, and is affected by X-ray magnification, radiography projection angle, and surveyor experience [30]. The robotic system can help to effectively improve these shortcomings. On the other hand, compared with other three-dimensional planning software, the used robotic system shows good preoperative planning ability. Inoue et al. used the 3D planning software Zed-hip for preoperative planning, and the complete accuracy of acetabular cup and femoral stem planning was 92% and 65%, respectively [31]. Huo et al. used artificial intelligence (AI) technology, AI HIP, for preoperative planning, and the complete accuracy of acetabular cup and femoral stem was 71.19% and 76.27%, respectively [32]. This study shows that the preoperative planning using the robotic system is more accurate than the traditional template measurement, which preliminarily confirms its accuracy and clinical application value.

The placement of prostheses in traditional THA mainly depends on the experience of the operators. We assume that the placement of acetabular prostheses can be better guided by rTHA. In our results, we use the angular difference of the acetabular cup between the actual and predicted values to comprehensively reflect the ability of preoperative planning and intraoperative decision-making of our robot. Our results show that the inclination/anteversion differences contained a similar trend, that is, rTHA is more often located in groups (< 6°), while cTHA is more widely distributed across all four groups. This implies that the actual angles deviate more from the planned angles in cTHA, suggesting that rTHA can reduce the placement error of the prosthesis and make the prosthesis reach the desired position as much as possible. Accurate matching between the prosthesis and the patient can be realized in three-dimensional view intraoperatively, and the error caused by the measuring instrument and the human can be effectively reduced. The robotic system can identify the surgical situation accurately, adjust in a timely manner, and finally make correct intraoperative decision-making during the operation.

The ideal leg length difference after THA is less than 1 cm, while conversely, it will lead to a series of complications, such as scoliosis, low back pain, and worn prostheses [29]. Traditionally, to avoid unequal lengths of the lower limbs and improve satisfaction after surgery, it is necessary to carry out careful preoperative examinations and use correct surgical techniques to judge whether the lower extremities are equal in time. There are many methods to balance the length of the lower extremities in THA, such as the Shuck test, knee comparative test of length, and Drop-Kick test [33]. However, these methods are affected by anesthesia, muscle relaxant dosage and subjective feelings of surgeons, affecting the accuracy of judgment. THA assisted by a manipulator can select the appropriate prosthesis through preoperative planning and adjust the depth and position of the prosthesis according to the feedback of the navigation system to reduce the unequal length difference of the lower extremities [34], reflecting the strong ability of preoperative planning and intraoperative decision-making.

The study has some limitations. First, as a prospective study, this trial selected only 60 patients, a small number of cases, and requires a larger sample to obtain clearer results. Second, although the robot planning navigation system was compared with the traditional X-ray template, this trial did not use a 3D template as a control. Third, CT scanning was performed by a robot planning navigation system before the operation, whose radiation dose and economic cost increase compared with the control group. Fourth, although there was no significant difference in BMI between the two groups, the closeness to significant difference may introduce some bias. Fifth, while the surgeons made efforts to adhere to the initial plan during the surgical procedure, it is possible that adjustments were made to the implant positions based on intraoperative findings and considerations, for both groups. This could introduce a slight margin of error in the experiment. Sixth, during preoperative planning, the rTHA group utilized anatomical inclination/anteversion angles for their planning, while the cTHA group used radiographic inclination/anteversion angles. This difference in approach is a result of the designed system differences and introduces potential bias to the experiment.

## Conclusion

Robotic arm-assisted total hip arthroplasty decreases leg length discrepancy in the lower extremities and exhibits comparable predictive effects regarding prosthesis size and placement to those of experienced orthopedic surgeons.

## References

[1] Ferguson RJ, Palmer AJ, Taylor A, Porter ML, Malchau H, Glyn-Jones S (2018). Hip replacement. Lancet.

[2] Kurtz S, Ong K, Lau E, Mowat F, Halpern M (2007). Projections of primary and revision hip and knee arthroplasty in the United States from 2005 to 2030. J Bone Jt Surg Am.

[3] Yan L, Ge L, Dong S, Saluja K, Li D, Reddy KS, Wang Q, Yao L, Li JJ, Roza da Costa B (2023). Evaluation of comparative efficacy and safety of surgical approaches for total hip arthroplasty: a systematic review and network meta-analysis. JAMA Netw Open.

[4] Huang K, Wu T, Lou J, Wang B, Ding C, Gong Q, Rong X, Liu H (2023). Impact of bone-implant gap size on the interfacial osseointegration: an in vivo study. BMC Musculoskelet Disord.

[5] Naito Y, Hasegawa M, Tone S, Wakabayashi H, Sudo A (2021). The accuracy of acetabular cup placement in primary total hip arthroplasty using an image-free navigation system. BMC Musculoskelet Disord.

[6] Gallo MC, Chung BC, Tucker DW, Piple AS, Christ AB, Lieberman JR, Heckmann ND (2021). Limb length discrepancy in total hip arthroplasty: is the lesser trochanter a reliable measure of leg length?. J Arthroplasty.

[7] Bono JV (2004). Digital templating in total hip arthroplasty. J Bone Jt Surg Am.

[8] Eggli S, Pisan M, Muller ME (1998). The value of preoperative planning for total hip arthroplasty. J Bone Jt Surg Br.

[9] de Thomasson E, Mazel C, Guingand O, Terracher R (2002). Value of preoperative planning in total hip arthroplasty. Rev Chir Orthop.

[10] Karam JA, Tokarski A, Deirmengian C, Thalody H, Kwan SA, McCahon J, Lutz R, Courtney PM, Deirmengian GK (2023). A video teaching tool is effective for training residents in hip arthroplasty templating. Cureus.

[11] Mittag F, Ipach I, Schaefer R, Meisner C, Leichtle U (2012). Predictive value of preoperative digital templating in THA depends on the surgical experience of the performing physician. Orthopedics.

[12] Carter LW, Stovall DO, Young TR (1995). Determination of accuracy of preoperative templating of noncemented femoral prostheses. J Arthroplasty.

[13] Levine B, Fabi D, Deirmengian C (2010). Digital templating in primary total hip and knee arthroplasty. Orthopedics.

[14] Unnanuntana A, Wagner D, Goodman SB (2009). The accuracy of preoperative templating in cementless total hip arthroplasty. J Arthroplasty.

[15] Whiddon DR, Bono JV, Lang JE, Smith EL, Salyapongse AK (2011). Accuracy of digital templating in total hip arthroplasty. Am J Orthop (Belle Mead NJ).

[16] Archibeck MJ, Cummins T, Tripuraneni KR, Carothers JT, Murray-Krezan C, Hattab M, White RE (2016). Inaccuracies in the use of magnification markers in digital hip radiographs. Clin Orthop Relat Res.

[17] Weber M, Woerner ML, Springorum HR, Hapfelmeier A, Grifka J, Renkawitz TF (2014). Plain radiographs fail to reflect femoral offset in total hip arthroplasty. J Arthroplasty.

[18] Tsai TY, Dimitriou D, Li G, Kwon YM (2014). Does total hip arthroplasty restore native hip anatomy? Three-dimensional reconstruction analysis. Int Orthop.

[19] Nakamura N, Nishii T, Kitada M, Iwana D, Sugano N (2013). Application of computed tomography-based navigation for revision total hip arthroplasty. J Arthroplasty.

[20] Sariali E, Mouttet A, Pasquier G, Durante E, Catone Y (2009). Accuracy of reconstruction of the hip using computerised three-dimensional pre-operative planning and a cementless modular neck. J Bone Jt Surg Br.

[21] Bosker BH, Verheyen CCPM, Horstmann WG, Tulp NJA (2007). Poor accuracy of freehand cup positioning during total hip arthroplasty. Arch Orthop Trauma Surg.

[22] Bullock EKC, Brown MJ, Clark G, Plant JGA, Blakeney WG (2022). Robotics in total hip arthroplasty: current concepts. J Clin Med.

[23] Murray DW (1993). The definition and measurement of acetabular orientation. J Bone Jt Surg Br.

[24] Wang RY, Xu WH, Kong XC, Yang L, Yang SH (2017). Measurement of acetabular inclination and anteversion via CT generated 3D pelvic model. BMC Musculoskelet Disord.

[25] Woolson ST, Hartford JM, Sawyer A (1999). Results of a method of leg-length equalization for patients undergoing primary total hip replacement. J Arthroplasty.

[26] Whittaker RK, Hexter A, Hothi HS, Panagiotidou A, Bills PJ, Skinner JA, Hart AJ (2014). Component size mismatch of metal on metal hip arthroplasty: an avoidable never event. J Arthroplasty.

[27] Wei JC, Li D, Sing DC, Yang J, Beeram I, Puvanesarajah V, Della Valle CJ, Tornetta P, Fritz J, Yi PH (2022). Detecting total hip arthroplasty dislocations using deep learning: clinical and Internet validation. Emerg Radiol.

[28] Barrow JA, Divecha HM, Panchani S, Boden R, Porter ML, Board TN (2019). Does oversizing an uncemented cup increase post-operative pain in primary total hip arthroplasty?. Eur J Orthop Surg Traumatol.

[29] Townsend S, Kim SE, Pozzi A (2017). Effect of stem sizing and position on short-term complications with canine press fit cementless total hip arthroplasty. Vet Surg.

[30] Mirghaderi SP, Sharifpour S, Moharrami A, Ahmadi N, Makuku R, Salimi M, Mortazavi SMJ (2022). Determining the accuracy of preoperative total hip replacement 2D templating using the mediCAD (R) software. J Orthop Surg Res.

[31] Inoue D, Kabata T, Maeda T, Kajino Y, Fujita K, Hasegawa K, Yamamoto T, Tsuchiya H (2015). Value of computed tomography-based three-dimensional surgical preoperative planning software in total hip arthroplasty with developmental dysplasia of the hip. J Orthop Sci.

[32] Huo JB, Huang GX, Han D, Wang XJ, Bu YF, Chen Y, Cai DZ, Zhao C (2021). Value of 3D preoperative planning for primary total hip arthroplasty based on artificial intelligence technology. J Orthop Surg Res.

[33] Tanino H, Sato T, Nishida Y, Mitsutake R, Ito H (2018). Hip stability after total hip arthroplasty predicted by intraoperative stability test and range of motion: a cross-sectional study. BMC Musculoskelet Disord.

[34] Manzotti A, Cerveri P, De Momi E, Pullen C, Confalonieri N (2011). Does computer-assisted surgery benefit leg length restoration in total hip replacement? Navigation versus conventional freehand. Int Orthop.

